# Differential Relationships of Child Anxiety and Depression to Child Report and Parent Report of Electronic Media Use

**DOI:** 10.1007/s10578-019-00892-7

**Published:** 2019-05-06

**Authors:** Payton Q. Fors, Deanna M. Barch

**Affiliations:** 1 Department of Psychological & Brain Sciences, Washington University, Campus Box 1125, 1 Brookings Drive, St. Louis, MO 63130 USA; 2 Department of Psychiatry, Washington University, St. Louis, MO USA; 3 Department of Radiology, Washington University, St. Louis, MO USA

**Keywords:** Children, Anxiety, Depression, Electronic media use, Technology

## Abstract

Child depression and anxiety have been associated with electronic media use, but the comorbidity between the two has rarely been accounted for in analyses. We examined both child and parent reports of electronic media use in relation to parent-reported child depression and anxiety. Using survey and interview data collected for 9- to 11-year-olds from the 21-site adolescent brain cognitive development study, we conducted generalized linear mixed models. Our results demonstrated that electronic media use was more strongly associated with depression than anxiety, and that accounting for depression significantly reduced the relationship between electronic media use and anxiety. Different categories of electronic media showed differential relationships to anxiety and depression, with video gaming and video chatting related to anxiety, but video watching related to depression. These findings provide important data to ground theories of the mechanisms that contribute to these associations.

**Electronic supplementary material:**

The online version of this article (10.1007/s10578-019-00892-7) contains supplementary material, which is available to authorized users.

## Introduction

Electronics are undisputedly increasingly pervasive in peoples’ daily lives. This is especially true for children; 95% of U.S. teens say they own or at least have access to a smartphone, and 88% a computer [1]. A multi-wave study by the Kaiser Foundation found that from 1999 to 2009, child daily media use increased by 69 min, and media exposure increased by over 3 h [2]. Although there are benefits of electronics for children, potential negative associations with childhood mental health are an emerging topic of interest in current research. There is some evidence that greater child anxiety is associated with higher overall levels of general electronic media use [3–6], as well as with greater use of specific types of electronics, such as video games [5, 7–9], and social media [9–12]. However, prior research has not examined whether such associations with anxiety remain significant after controlling for depression, which is often comorbid with anxiety. Further, it is not clear whether parent or child reports of electronic media use are equally associated with child mental health. The goal of the current study was to address these questions.

The definition of electronic media use (EMU) varies across the extant literature. General EMU studies typically include total computer use and television use, and somewhat less commonly total cellphone use and tablet use in their definitions. The American Academy of Pediatrics, when discussing “screen time,” counts television, movies, social media, and video games in their definition [13]. As another example, Lange et al. included television, computer/internet, video games, total screen time, mobile phones, and music in their definition of EMU [14]. Of note, most studies are not focused on the media content, but rather are on the time spent using electronic media. These variations in definition of EMU need to be considered when reviewing extant literature on EMU and will be noted in descriptions of studies below.

A body of research has shown EMU to be associated with degradation of a number of different aspects of childhood health. For example, greater EMU has been associated with reduced quantity and quality of sleep, [14, 15], heavier weight [16], and higher metabolic risk scores, calculated from cholesterol levels, hemoglobin levels, waist size, and various measures of blood pressure, [17]. Greater EMU has also been associated with nonphysical measures of health, such as children’s life satisfaction [9, 18], and their mental health [6]. In particular, previous studies have found positive correlations between EMU and anxiety levels. Maras et al. analyzed cross-sectional data on Canadian youth and found a positive association between EMU duration (television, computer, and video game use across weekdays and weekends) and anxiety severity. Cao et al. also found a positive correlation between general EMU (television use and computer use) and anxiety in their study of Chinese adolescents [3]. Other studies have found correlations between “Problematic Technology Use” and anxiety [5], and between general EMU and anxiety over a 4 year span [4]. Thus, the literature suggests that there is an association between greater EMU and higher anxiety in children, though the direction of causality remains unclear.

Relatedly, other studies have found positive associations between child EMU and depression [6]. Twenge et al. [19] found that depression and the time spent using electronics were positively associated in annual nationally representative surveys, with these relationships increasing over a six-year span of such surveys, starting in 2009. A meta-analysis by Liu et al. came to similar conclusions, finding a positive association between electronic use and depression [20]. However, importantly, none of the studies described above addressed the issue of comorbidity between anxiety and depression, which is common [21–23]. More specifically, most studies did not control for depression when examining relationships to anxiety, nor did they control for anxiety when examining relationships to depression. Thus, an open question is whether there are unique relationships between either anxiety or depression and EMU that are not accounted for by comorbidity with the other.

This research raises the question of why anxiety and/or depression might be associated with EMU. One possibility is that individuals with anxiety and/or depression use EMU to cope with or avoid their negative emotional experiences. Anxiety and depression have repeatedly been linked to people’s levels of experiential avoidance, defined as the effort to suppress unwanted emotions and experiences, while not changing the emotions and experiences themselves. For example, Epkins defines experiential avoidance as involving “engaging in emotion regulation strategies or behaviors aimed to suppress, avoid, or escape undesirable thoughts, emotions, physical bodily sensations, or other unpleasant private experiences” [24]. Venta et al. found an association between experiential avoidance and anxiety in inpatient adolescents, with anxiety associated with experiential avoidance even when controlling for depression [25]. Epkins also found that experiential avoidance correlated with child depression, as well as with anxiety and social anxiety even when controlling for depression [24]. This link between anxiety and avoidance raises the possibility that EMU could be a way that anxious children, and potentially also depressed children, engage in experiential avoidance.

Different categories of EMU may also show distinct relationships to anxiety versus depression, either overall, or within specific genders, though findings have been mixed. For example, research has shown a relationship between video gaming and anxiety in both males and females [8, 9], but other work found this relationship only in males [7], and other work did not find such a relationship in either sex [5]. Other research has examined social media usage, which has been associated with anxiety [9, 12], anxiety predicted from social media fatigue (defined as mental exhaustion after digital information and interaction overload) [11], and poorer well-being, though in some studies only in females [10]. A survey conducted by the Royal Society for Public Health found that, on average, adolescents reported increased anxiety and depression after using Facebook, Twitter, Snapchat, and Instagram [26]. These findings are consistent with the hypothesis that social media use could be contributing to increases in anxiety or depression symptoms, potentially through social comparison [12, 27]. More research is needed to examine specific types of electronic media use in relationships to child anxiety and/or depression.

Another open issue is whether parent or child reports of EMU differentially predict anxiety or depression. The majority of research asks children for report of EMU, compared to parents. Studies have found relatively modest correlations between parent and child report of EMU [28, 29] and research has mixed results in who reports more EMU [28–32]. Lower reports by parents may reflect parents perceiving that they have greater control over their child’s EMU than they actually do. In these cases, it is also possible that children inflate their report of EMU so as to boost their social standings among their peers. Tweens tend to report that their parents know “a lot” about their media use more than teens do, suggesting that age may factor into report discrepancies [33]. If parent report tends to underestimate EMU in children, it is possible that child report will be more strongly related to anxiety and/or depression levels.

As described above, there is growing evidence that overall EMU is associated with a variety of negative child mental health outcomes. However, there are a number of open questions in the literature in regard to the relationship between EMU and child mental health. The goal of the current analyses was to address the following questions using data from a large-scale study of 9- to 11-year-old children in the United States—the Adolescent Brain Cognitive Development (ABCD) Study. First, we asked whether there was a relationship between overall EMU and anxiety in the ABCD Study, to determine whether we could replicate previous findings in the literature of such a relationship. Second, we asked whether there was a relationship between overall EMU and depression, and whether the relationships of EMU and anxiety held if we controlled for depression. We predicted that overall EMU would remain related to anxiety even when controlling for depression if anxious children used EMU as a coping mechanism. Third, we also asked whether there were any differential relationships of child versus parent report of EMU to anxiety and/or depression. We predicted that child report would be more strongly related to anxiety than parent report, given the evidence reviewed above about parent’s potential underreporting child EMU use. Lastly, we examined whether there were differential relationships of specific types of EMU to anxiety or depression, and whether there were any gender differences in these relationships. Given the existing literature described above, we expected that the video gaming and social networking categories of EMU would be the strongest predictors of anxiety or depression symptoms compared to television, movie watching, video watching (e.g. YouTube), texting, and video chatting.

## Methods

### Participants

Data from a sample of 4524 children in the ABCD study were used [34–36]. Due to missing values on some measures, the sample size for the current analysis was 4139 children. Participants were excluded from analysis if they or their parent/guardian did not complete a questionnaire, or if they answered, “do not know” for questions. The included participants were 8.9–11 years of age (mean = 10 years, SD = 0.61), and were close to evenly distributed between sexes (1977 girls and 2162 boys). 15.2% of children were Black American, and 8.2% were of other minority ethnic backgrounds. Written and verbal consent were collected from all parents/guardians, and written and verbal assent from all children were collected as well. The data used were obtained through the National Database for Autism Research through the National Institute of Mental Health Data Archive.

### Measures

#### Screen Time

The ABCD Screen Time Survey (STQ) was completed by the children, and a different, shorter STQ was completed by the child’s parent/guardian [37]. The STQ for children is a 14-item questionnaire that asks how many hours per day a child uses different forms of electronic media. The responses range from “0 h” (0) to “4 + h” (4) and the questionnaire asks about 6 forms of media on weekdays and on weekends. These media include: television shows and movies, videos, video games, texting, social networking, and video chatting. The last two questions of the youth STQ pertain to the frequency of mature video game consumption and R-rated movie consumption. These two questions were not used in analyses. Total weekday and weekend minutes for screen time based on child reports were calculated by adding the total hours reported for weekday use or weekend use, and then multiplying by 60. The STQ for parents is a 2-item questionnaire that asks about the child’s total media use on weekdays and on weekends, as a continuous variable in minutes. The ABCD Screen Time Questionnaires were based on measures developed by Sharif et al. [38]. For parent reports of EMU, EMU is defined based on devices, specifically on a “computer, cellphone, tablet, or other electronic device” (Jernigan et al. [35, 36].

#### Anxiety

Parents/guardians completed the Child Behavior Checklist (CBCL), a 112-item questionnaire that asks about various symptoms and behaviors the adolescent may exhibit [37, 39]. The CBCL contains 9 statements used to form an anxiety subscale of the CBCL. The subscale includes statements such as “worries” and “self-conscious or easily embarrassed.” Responses were scaled from 0 (not true) to 2 (very true/often true). T-scores were calculated and used for analysis. A T-score of 65 was considered to be in the clinical range for anxiety.

#### Depression

The CBCL was also used to measure depression symptoms in children [37, 39]. A subscale containing 13 statements, such as “There is very little he/she enjoys” and “Underactive, slow moving, or lacks energy,” each scaled from 0 to 2. T-scores were calculated and used for analysis. A T-score of 65 was considered to be in the clinical range for depression.

#### Physical Activity

EMU could be related to child anxiety or depression because it is a proxy for reduced physical activity, which has also been linked to mental health [3, 4]. To control for this, we utilized a measure of child reported physical activity. Children were asked “During the past 7 days, on how many days were you physically active for a total of at least 60 min per day? (Add up all the time you spent in any kind of physical activity that increased your heart rate and made you breathe hard some of the time).” The wording of the question aimed to make clear that participants could include both organized activities (such as gym class or an extracurricular sport), and individual activities. The wording also permitted participants to consider that the 60 min did not have to occur all at once. Answers could range from 0 to 7.

#### Body Mass Index (BMI)

Similar to physical activity, EMU could be related to child anxiety or depression because it is a proxy for increased BMI, which has also been linked to mental health [40, 41]. Height was measured up to three times, and the average of the two closest heights was taken (or of all three if one of the values was equidistant from the other two). The same was done to measure weight. BMI was then calculated using the average height and average weight of each adolescent.

#### Income

Total family income was determined by asking the parent the total combined family income before taxes for the previous 12 months. The income was then scaled from less than $5000 each year (1) to $200,000 or more each year (10). In terms of family income, 25% of the sample had incomes at or below $49,999, 30.3% had incomes between $50,000 and $99,999, and 44.7% had incomes above $100,000.

### Data Analysis

Analyses were carried out using IBM SPSS Statistics, Version 25. First, the interrelationships among the measures of EMU were examined using Pearson’s Product Moment Correlations. To compare mean values on the child and parent reports of EMU, ANOVA was used. All remaining analyses used generalized linear mixed models, and accounted for nesting within testing site and family, and controlled for age, sex, BMI, family income, race, and physical activity. We first examined the relationship between each measure of EMU and anxiety symptoms. Then, parallel analyses predicting depression were conducted. The analyses predicting anxiety were then repeated, controlling for depression. The reverse analyses were also carried out, predicting depression, controlling for anxiety. Relationships to specific types of EMU were also analyzed using generalized linear mixed models. As with general EMU, four sets of analyses were run: predicting anxiety, predicting depression, predicting anxiety while controlling for depression, and predicting depression while controlling for anxiety. As a final comparison of EMU reports, all four general EMU measures were entered into one generalized linear mixed model for anxiety, then for depression, then for anxiety controlling for depression, and finally for depression controlling for anxiety. The significance level for all tests was 0.05. Sex effects were considered by adding an effect for the interaction of the EMU measure and sex. For any model with a significant effect for this interaction, a generalized linear mixed model was computed for each sex separately. Of note, we standardized all variables in the analyses presented below to allow clearer interpretation of the coefficients in terms of effect sizes.

## Results

### Comparison of Mean Levels and Associations

Table 1 provides the means, standard deviations, and range of values for variables used in the analyses reported below. First, we examined the interrelationships between the two child report EMU measures and the two parent report EMU measures, using the general linear models described in the methods that controlled for age, sex, BMI, family income, race, and physical activity. The two child report measures of EMU (*β *=0.71*, t* = 67.68*, p *<0.001) and the two parent report measures (*β *=0.48, *t* = 34.74*, p *<0.001) were highly correlated. The child and parent report measures of both weekday (*β *=.18, *t* = 12.26*, p *<0.001) and weekend (*β *=0.16, *t* = 12.06*, p *<0.001) EMU were also correlated. Next, we compared the magnitude of use reported by parents versus children, using a repeated measures ANOVA with day (weekday versus weekend) and source (parent versus child) as within-subject factors. There were main effects of day (*F (1,4500*) = 2579.58*, p *=< 0.001) and source (*F (1,4500) *= 255.92*, p* < 0.001), and an interaction between day and source (*F (1,4500) *= 91.56*, p *< 0.001). As can be seen from the values in Table 1, children reported overall more EMU than parents, and both children and parents reported more EMU on the weekends than the weekdays. However, the interaction reflected that the degree to which children reported greater EMU than parents was larger on weekdays versus weekends.

**Table 1:** Sample characteristics

Measure	Mean	Standard deviation	Minimum	Maximum
Anxiety	53.37	5.939	50	94
Depression	53.43	5.57	50	89
Kid weekday EMU (min)	197.89	175.40	0.00	1440.00
Kid weekend EMU (min)	263.02	205.17	0.00	1440.00
Parent weekday EMU (min)	140.29	140.34	0.00	1440.00
Parent weekend EMU (min)	232.33	149.05	0.00	1440.00
Types of weekday electronic media use (child report)				
TV shows, movies	67.42	1.10	0.00	240.00
Videos	49.48	1.11	0.00	240.00
Video games	54.48	1.11	0.00	240.00
Texting	11.99	0.51	0.00	240.00
Social networks	5.55	0.36	0.00	240.00
Video chatting	9.04	0.41	0.00	240.00
Types of weekend electronic media use (child report)				
TV shows, movies	97.08	1.25	0.00	240.00
Videos	62.23	1.26	0.00	240.00
Video games	71.76	1.28	0.00	240.00
Texting	13.86	0.60	0.00	240.00
Social networks	6.97	0.45	0.00	240.00
Video chatting	11.21	0.52	0.00	240.00

### Relationships of Overall EMU to Anxiety and Depression

All generalized linear mixed models used age, sex, BMI, family income, race, and physical activity as covariates, and accounted for testing site and family nesting of individuals. We initially examined the relationships of child anxiety and child depression to overall EMU, as shown in Table 2. Child report of weekday EMU and both child and parent report of weekend EMU were significantly associated with anxiety symptoms. For depression, both child and parent report of both weekday and weekend EMU were significantly associated with depression. We next asked whether either child or parent reported EMU continued to be associated with anxiety after controlling for depression. No EMU measure showed a significant relationship with anxiety symptoms when controlling for depression. All reports of EMU remained significantly related to depression when controlling for anxiety, other than child report of weekday EMU. Of note, while many of the associations were significant, the effect sizes were relatively small.

**Table 2:** Relationships between anxiety or depression and electronic media use

Variable	Coefficient	t-value	Sig.	− 95% CI	+ 95% CI
Anxiety					
Child reported weekday use	0.055	3.309	0.001	0.022	0.088
Child reported weekend use	0.060	3.689	< 0.001	0.028	0.092
Parent reported weekday use	0.025	1.558	0.119	− 0.006	0.057
Parent reported weekend use	0.070	4.255	< 0.001	0.038	0.102
Depression					
Child reported weekday use	0.050	3.026	0.002	0.018	0.082
Child reported weekend use	0.066	4.095	< 0.001	0.034	0.098
Parent reported weekday use	0.044	2.740	0.006	0.013	0.076
Parent reported weekend use	0.084	5.158	< 0.001	0.052	0.116
Anxiety accounting for depression					
Child reported weekday use	0.026	1.955	0.051	− 0.000	0.052
Child reported weekend use	0.021	1.609	0.108	− 0.005	0.047
Parent reported weekday use	− 0.001	− 0.062	0.950	− 0.026	0.024
Parent reported weekend use	0.021	1.631	0.103	− 0.004	0.047
Depression accounting for anxiety					
Child reported weekday use	0.018	1.346	0.178	− 0.008	0.044
Child reported weekend use	0.032	2.427	0.015	0.006	0.057
Parent reported weekday use	0.029	2.251	0.024	0.004	0.054
Parent reported weekend use	0.042	3.251	0.001	0.017	0.068

### Relationships of Parent Versus Child and Weekday Versus Weekend EMU to Anxiety and Depression

We next conducted a generalized linear mixed model with all four EMU reports as predictors of anxiety, and the same covariates described above. In this model, parent weekend report of general EMU was a significant independent predictor of anxiety (*t *=3.43*, p *=0.001). However, when depression was added as a covariate, none of the EMU measures significantly predicted anxiety. For depression, child weekend EMU report and parent weekend EMU report were each significantly related to depression (*t *= 2.38, *p* = 0.017 and *t *= 3.79, *p* < 0.001 respectively), while neither weekday EMU report had significant relation to depression. When anxiety was added as a covariate, parent weekend EMU continued to be a significant independent predictor (*t *= 2.13, *p* = 0.034).

### Sex Differences

We analyzed whether sex moderated any of the relationships between EMU report and either anxiety or depression. The only significant interaction with sex was for child report of weekend EMU predicting anxiety (*t* = − 3.51, *p* < .001). Looking at separate mixed models for boys and girls, boys’ weekend EMU was significantly associated with anxiety symptoms (*t* = 4.88, *p *< .001), but girls’ weekend EMU was not (Table 3). For depression, no measures of EMU demonstrated sex differences.

**Table 3:** Weekend EMU in relationship to anxiety by sex

Variable	Coefficient	t-value	Sig.	− 95% CI	+ 95% CI
Boys					
Overall use	0.109	4.880	< 0.001	0.065	0.153
TV shows, movies	0.072	3.234	0.001	0.028	0.115
Videos	0.074	3.388	0.001	0.031	0.116
Video games	0.054	2.517	0.012	0.012	0.095
Texting	0.081	3.418	0.001^a^	0.034	0.127
Social networks	0.061	2.574	0.010	0.015	0.108
Video chatting	0.069	3.131	0.002^a^	0.026	0.113
Girls					
Overall use	0.001	0.047	0.962	− 0.045	0.047
TV shows, movies	− 0.002	− 0.084	0.933	− 0.045	0.041
Videos	0.013	0.555	0.569	− 0.033	0.059
Video games	− 0.007	− 0.272	0.786	− 0.060	0.045
Texting	− 0.013	− 0.656	0.512	− 0.054	0.027
Social networks	0.003	0.168	0.867	− 0.037	0.044
Video chatting	0.008	0.361	0.718	− 0.035	0.050

^a^Still significant when controlling for depression

### Depression and Anxiety as a Function of EMU

In addition, to examine whether the percentages of children who met “clinical” cutoffs on the depression and anxiety measures (T scores of 65 or above) differed as a function of EMU, we conducted additional analyses using Chi Square tests to examine percentage of children above the clinical cutoff for depression and anxiety as a function of amount of EMU. The range of electronic media use varied on weekdays versus the weekend. Thus, for weekday (both parent and child reports), we grouped EMU by 0 to 60 min (up to 1 h), 60 to 120 min (1 to 2 h), 120 to 180 min (2 to 3 h) and greater than 180 min (more than 3 h). For weekend (both parent and child reports), we grouped EMU by 0 to 120 min (up to 2 h), 120 to 240 min (2 to 4 h), 240 to 360 min (4 to 6 h) and greater than 360 min (more than 6 h). As shown in Supplemental Table 3, the Chi Square analysis was significant for every combination of category of EMU report and both depression and anxiety in the total sample. In the vast majority of cases, the percentage of children above the clinical cutoffs for both anxiety and depression was close to double (or higher) between the lowest category of EMU and the highest category of EMU for both child and parent report and for both anxiety and depression, with a relatively linear increase across levels of EMU. As noted above, the only interaction with sex and EMU we found was for weekend EMU predicting anxiety. As shown in Supplemental Table 4, for boys, the Chi Square analysis was again significant for every combination of category of EMU report and both depression and anxiety, again with a close to doubling of percentage of boys above the clinical cutoffs in the highest versus lowest EMU use categories. As shown in Supplemental Table 5, for girls, the patterns were similar for depression for both parent and child reported EMU, with significant Chi Squares for both parent EMU reports, but with overall lower levels of depression. For anxiety, the patterns were more variable with the second highest EMU level showing increases in percentages of children above clinical cutoffs in most cases, but the highest levels sometimes dropping down again. These different patterns for boys and girls for anxiety were consistent with the primary results presented in the text.

### Relationships of Specific Types of EMU to Anxiety and Depression

We next examined relationships of different categories of EMU to anxiety and depression. Both weekday and weekend video watching were significantly associated with anxiety symptoms (Table 4), though only weekday video watching remained significant when controlling for depression (Supplemental Table 1). Video games use significantly associated with anxiety on the weekdays and trend level associated on the weekends, but neither relationship remained significant when controlling for depression. Weekday, but not weekend, social media uses was associated with anxiety, while weekend, but not weekday, video chatting and TV show/movie watching was significantly associated with anxiety. Weekend video chatting remained significantly associated with anxiety even when controlling for depression.

**Table 4:** Categories of EMU in relationship to anxiety

Variable	Coefficient	t-value	Sig.	− 95% CI	+ 95% CI
Weekday					
TV Shows, movies	0.017	1.046	0.296	− 0.015	0.048
Videos	0.042	2.604	0.009	0.010	0.073
Video games	0.051	3.124	0.002^a^	0.019	0.083
Texting	0.021	1.320	0.187	− 0.010	0.051
Social networks	0.031	2.014	0.044	0.001	0.062
Video chatting	0.016	1.016	0.309	− 0.015	0.046
Weekend					
TV shows, movies	0.034	2.176	0.030	0.003	0.065
Videos	0.048	3.004	0.003	0.017	0.079
Video games	0.032	1.914	0.056	− 0.001	0.064
Texting	0.030	1.908	0.056	− 0.001	0.060
Social networks	0.029	1.870	0.062	− 0.001	0.060
Video chatting	0.038	2.487	0.013^a^	0.008	0.069

^a^Still significant when controlling for depression

Both weekday and weekend video watching and video gaming (Table 5) were significantly associated with depression, with the relationships to video watching remaining even when controlling for anxiety (Supplemental Table 2). On weekends, but not weekdays, television show and movie watching, was significantly associated with depression, but this relationship was no longer significant when covarying for anxiety. When examining further the interaction with sex for child report of weekend EMU, each of the 6 categorical measures of EMU showed the same patterns, with boys’ reports significantly associating with anxiety symptoms, but girls’ reports having no significant association (Table 3).

**Table 5:** Categories of EMU in relationship to depression

Variable	Coefficient	t-value	Sig.	− 95% CI	+ 95% CI
Weekday					
TV shows, movies	0.026	1.643	0.101	− 0.005	0.057
Videos	0.049	3.088	0.002^a^	0.018	0.080
Video games	0.036	2.221	0.026	0.004	0.068
Texting	0.011	0.685	0.493	− 0.020	0.041
Social networks	0.021	1.339	0.181	− 0.010	0.051
Video chatting	0.000	0.018	0.986	− 0.030	0.031
Weekend					
TV shows, movies	0.039	2.494	0.013	0.008	0.069
Videos	0.076	4.812	< 0.001^a^	0.045	0.107
Video games	0.034	2.061	0.039	0.002	0.066
Texting	0.021	1.330	0.184	− 0.010	0.051
Social networks	0.017	1.101	0.271	− 0.013	0.047
Video chatting	0.016	1.053	0.292	− 0.014	0.046

^a^Still significant when controlling for anxiety

## Discussion

This study examined the relationship between EMU separately with anxiety and depression. Both anxiety and depression related to all or almost all measures of EMU, whether from parent report or child report. However, when controlling for depression, none of the EMU reports remained significantly associated with anxiety. In contrast, most of the depression associations remained when controlling for anxiety; only child weekday EMU lost its association with depression. In addition, we found that parent weekend EMU report independently predicted depression even when controlling for other EMU reports and anxiety. When looking at specific types of EMU, we found that video gaming and video chatting had the most robust associations with anxiety. In contrast, video watching had the most robust associations with depression. We also found an interaction with sex for child report of weekend EMU use predicting anxiety, with significant relationships for boys, but not girls. Each of these findings will be discussed in more detail below.

As expected, we found a significant relationship between EMU and anxiety for three of the four general EMU reports. These results fit well with prior research demonstrating an association between anxiety and EMU [3–6]. Further, we also found significant relationships between depression and all four EMU measures, again consistent with previous literature [3, 6, 19, 20]. However, after accounting for the comorbidity of depression with anxiety, none of the EMU reports remained significantly associated with anxiety. As such, our results suggest that the interpretation of findings from previous studies linking EMU to anxiety may need to be re-evaluated. The majority of these previous studies did not control for depression when examining relationships to anxiety [3–6], and thus it is possible that their findings are more reflective of a relationship between EMU and depression, given the frequent comorbidity of anxiety and depression.

In contrast, three of the EMU relationships with depression remained significant even when controlling for anxiety. These results suggest a stronger relationship between EMU and depression compared to anxiety. Our findings are consistent with the body of literature linking EMU to depression in both children and adolescents [6, 19, 20]. This relationship could suggest that children with depression are using EMU to cope with negative feelings. Alternatively (or in addition), a child’s depression could interfere with them engaging in non-EMU activities, particularly on the weekends, with EMU potentially becoming a default activity used to occupy their time. This explanation would be in line with the concept of experiential avoidance, again as described in the introduction [24, 25]. These hypotheses suggest the possibility that modification of EMU might be a useful part of interventions designed to target child depression. EMU interventions, such as limiting time spent on electronics and encouragement of prosocial activities, might be effectively targeted for children at risk for depression, though it is less clear whether this would be as effective for anxiety. However, more evidence about the causal relationship between EMU and depression or anxiety is needed; if EMU is an outcome rather than a contributor to depression or anxiety, modifying EMU may have limited positive impact.

Of the four general EMU reports, parent weekend EMU report was the only significant independent predictor of depression, after accounting for anxiety. It is not entirely clear why parent weekend EMU report was an independent predictor of depression. It may be that the parent weekend EMU report captured something not captured in other measures. All measures of EMU were subjective, so it is possible that parent perception of weekend EMU and of child depression symptoms from the parent’s point of views correlated more strongly than other EMU measures. It could also be that it is more obvious when children are engaged in EMU on the weekends compared to weekdays, and parents may consider this EMU relative to engagement in other activities more than their children. Further research will be needed to confirm this particular strong relationship of parent reported EMU to depression, ideally with converging evidence from child as well as parent reported depression.

We found only very modest evidence of sex differences in the relationships between EMU and anxiety or depression. Child report of weekend EMU was more strongly associated with anxiety in boys than in girls, with no significant relationship in girls. The lack of significant relationships between girls’ use of electronics and their anxiety symptoms could indicate that girls use other methods to cope with anxiety, rather than using electronic media, if the relationship between EMU and anxiety in boys reflects a coping mechanism. If this finding were limited to the use of video games, it could reflect an overall greater engagement in video gaming by boys as compared to girls. However, we found this same pattern for all forms of EMU, even forms that are not more likely to be used by boys (e.g., texting). As for depression and EMU, the lack of sex differences could suggest that boys and girls at this age with depression symptoms do not use electronics in different ways to cope, or that depressive symptoms do not result from EMU in different ways for boys or girls.

As anticipated given previous literature [7–9], anxiety was associated with weekday video gaming even after controlling for depression. This could potentially mean that children with anxiety symptoms use video games to cope on the weekdays rather than engaging in in-person activities after school. However, we did not anticipate the significant association between weekend video chatting and anxiety, a relationship that remained even after controlling for depression. One speculative hypothesis is that anxious children may use video chatting on the weekends because interacting with people through a screen feels less stressful for them. However, we would need more information on the content of children’s video chatting to better understand the nature of this relationship. In terms of depression, after controlling for anxiety, only video watching on the weekdays and weekends remained associated with depression. While we did not predict this, as noted above, it could reflect the possibility that children with depression are passively watching videos rather than engaging with peers or family members in extracurriculars or other activities on weekdays or weekends.

We found a significant association between television show and movie watching on the weekends with both anxiety and depression. These associations were lost with the controlling of anxiety for depression and depression for anxiety, suggesting that this relationship reflects a more general psychological distress factor. However, this finding contrasts with some prior work, such as the study by Mathers et al., which did not find an association between television watching and psychological wellbeing [8]. Another unexpected finding was that child use of social media was only weakly associated with anxiety symptoms (associations that were lost when controlling for depression) and was not associated at all with depression symptoms. As noted in the introduction, some previous literature suggestions an association between social media use and anxiety [9, 12]. However, our findings of a lack of association may reflect the current age of the children, as they displayed low levels of social media use; the average time spent on social media was the lowest value of all types of EMU both on weekdays and on weekends (Table 1). A stronger relationship between anxiety and/or depression and social media use may emerge as the children move into adolescence.

Limitations of this study include its current cross-sectional nature. Since the ABCD study has only released baseline data, longitudinal analyses are not possible at this time. This prevents stronger examination of causal relationships between EMU and anxiety or depression, though the longitudinal nature of the ABCD study will allow for such analyses in the future. Also, our findings were based on surveys and not on objective measures of EMU. The use of self-report or parent report surveys may not always accurately capture EMU, as parents may not be fully aware of all of their child’s electronic media use and children may not always be accurate reporters. Additionally, the use of hours of reported screen time as a measure of EMU, rather than other indices such as frequency of EMU, may miss important aspects of children’s use of technology [42]; Rosen et al. [43]. For example, the ABCD surveys do not assess potentially simultaneous use of multiple forms of EMU, nor do they fully capture the specific locations and devices that children are using for media consumption. However, it is challenging to use more direct or objective measures of technology use in very large studies such as the ABCD, though there are workgroups focused on identifying potential ways to add more objective measures to the ABCD study in the future. Further, parent reported anxiety and/or depression may not fully capture a child’s emotion experience, as the parent may not have full access to a child’s level of depression or anxiety, since some children may not articulate their feelings to their parents. As for children, self-report on measures of electronic use are subject to bias if the child cannot accurately estimate their use, or if the child inflates or deflates their use for social esteem or to appear a better-behaved child. Further, while our findings were significant, the effect sizes were small, with standardized beta weights in the range of 0.05 to 0.1. Thus, while we did find significant associations, the magnitude of these associations in this large population-based sample were small.

Future studies could benefit from inquiring about the nature of electronic media content. This could increase the specificity about what types of electronic media are most likely to be associated with child anxiety or depression. For example, a study by Holtz and Appel found that fantasy game players were significantly more likely to report internalizing problems than non-fantasy game players [44]. Holtz and Appel also found that those who preferred first-person shooter games were more likely to report externalizing problems. These results support the notion of that the content of certain types of EMU may relate differentially to certain types of psychological issues. Another important avenue for future research would be to inquire about the social nature of children’s EMU. For example, the present study does not ask whether children were watching television and movies with others, or if they were playing multi-player games or playing with other children. EMU that occurs in a social context may have different relationships to children’s mental health compared to solitary EMU. In addition to these directions, as future waves of data from the ABCD become available it will be possible to examine trajectories of EMU, anxiety, and depression, as well as examine leading and lagging relationships that may help identify causal relationships. Observing trends for EMU and psychological symptoms over multiple years may help elucidate the direction of relationships between EMU and anxiety and/or depression. Further, we may also find that the relationship between EMU and anxiety and/or depression changes as children move through different developmental periods and as different types of EMU use change in frequency or availability in the same children used for this analysis. Indeed, there is some evidence that psychological problems can be more associated with categories of EMU for teens than for preteens [43].

## Summary

This study contributes to the growing body of research on electronic media use, anxiety symptoms, and depression symptoms in children. Our results suggest that in the pre-teen years, there is a stronger association between EMU and depression, than between EMU and anxiety, with evidence for gender differences in the relationship between EMU use and anxiety. Further, we find that certain types of EMU are more strongly related to mental health in children, though this may change as the children move into adolescence. The longitudinal analyses that will be supported by the additional future waves of assessment as part of the ABCD study will allow for further investigation of causal relationships and changing relationships between EMU and mental health that may emerge across the course of development.

## Electronic supplementary material

Below is the link to the electronic supplementary material.

Supplementary material 1 (DOCX 31 kb)
