# Supplementary material for: Differential Relationships of Child Anxiety and Depression to Child Report and Parent Report of Electronic Media Use
Source: Child Psychiatry Hum Dev. 2019 May 6;50(6):907–17. doi: 10.1007/s10578-019-00892-7 (PMC6790192; doi:10.1007/s10578-019-00892-7)
Supplement: Supplementary file 1 — Supplementary material 1 (DOCX 31 kb) [file 10578_2019_892_MOESM1_ESM.docx]

| Table S1: Categories of EMU in Relationship to Anxiety, Controlling for Depression | | | | | |
| --- | --- | --- | --- | --- | --- |
| Variables | Coefficient | t-value | Sig. | -95% CI | +95% CI |
| **Weekday** |  |  |  |  |  |
| TV Shows and Movies | .002 | .158 | .875 | -.023 | .027 |
| Videos | .013 | 1.042 | .297 | -.012 | .039 |
| Video Games | .031 | 2.315 | .021 | .005 | .056 |
| Texting | .015 | 1.183 | .237 | -.010 | .040 |
| Social Networks | .017 | 1.372 | .170 | -.007 | .042 |
| Video Chatting | .016 | 1.279 | .201 | -.009 | .041 |
| **Weekend** |  |  |  |  |  |
| TV Shows and Movies | .011 | .845 | .398 | -.014 | .035 |
| Videos | .003 | .242 | .808 | -.022 | .028 |
| Video Games | .012 | .914 | .361 | -.014 | .038 |
| Texting | .018 | 1.405 | .160 | -.007 | .042 |
| Social Networks | .018 | 1.432 | .152 | -.007 | .043 |
| Video Chatting | .031 | 2.486 | .013 | .007 | .056 |

| Table S2: Categories of EMU in Relationship to Depression, Controlling for Anxiety | | | | | |
| --- | --- | --- | --- | --- | --- |
| Variable | Coefficient | t-value | Sig. | -95% CI | +95% CI |
| Weekday | | | | | |
| TV Shows and Movies | .014 | 1.113 | .266 | -.011 | .039 |
| Videos | .025 | 1.973 | .049 | .000 | .050 |
| Video Games | .008 | .591 | .554 | -.018 | .034 |
| Texting | -.004 | -.316 | .752 | -.029 | .021 |
| Social Networks | .006 | .513 | .608 | -.018 | .031 |
| Video Chatting | -.008 | -.658 | .510 | -.033 | .016 |
| Weekend | | | | | |
| TV Shows and Movies | .019 | 1.531 | .126 | -.005 | .044 |
| Videos | .048 | 3.745 | <.001 | .023 | .073 |
| Video Games | .017 | 1.245 | .213 | -.010 | .043 |
| Texting | .002 | .138 | .890 | -.023 | .026 |
| Social Networks | .004 | .288 | .773 | -.021 | .028 |
| Video Chatting | -.007 | -.576 | .564 | -.032 | .017 |

**Table S3: Percentages of Children in Clinical Range for Depression and Anxiety as a Function of Electronic Media Use**

|  |  |  | **Clinical Depression** | | **Clinical Anxiety** | | **Clinical Depression** | | **Clinical**  **Anxiety** | |
| --- | --- | --- | --- | --- | --- | --- | --- | --- | --- | --- |
|  |  | **% in Screentime Category** | NO | YES | NO | YES | χ2 | χ2 *p* | χ2 | χ2 *p* |
| Parent Weekday EMU | 0-60 minutes | 32.07% | 95.20% | 4.80% | 94.40% | 5.60% | 24.18 | <0.001 | 14.04 | 0.003 |
|  | 60 to 120 minutes | 31.88% | 94.10% | 5.90% | 92.50% | 7.50% |  |  |  |  |
|  | 120 to 180 minutes | 18.24% | 91.40% | 8.60% | 90.20% | 9.80% |  |  |  |  |
|  | Greater than 180 minutes | 17.81% | 90.30% | 9.70% | 91.20% | 8.80% |  |  |  |  |
| Parent Weekend EMU | 0-120 minutes | 26.35% | 95.40% | 4.60% | 94.30% | 5.70% | 33.42 | <0.001 | 23.84 | <0.001 |
|  | 120 to 240 minutes | 41.82% | 93.80% | 6.20% | 92.90% | 7.10% |  |  |  |  |
|  | 240 to 360 minutes | 21.29% | 92.50% | 7.50% | 91.90% | 8.10% |  |  |  |  |
|  | Greater than 360 minutes | 10.54% | 87.40% | 12.60% | 87.20% | 12.80% |  |  |  |  |
| Child Weekday EMU | 0-60 minutes | 21.92% | 95.30% | 4.70% | 93.30% | 6.70% | 21.84 | <0.001 | 10.97 | 0.012 |
|  | 60 to 120 minutes | 24.09% | 94.70% | 5.30% | 94.40% | 5.60% |  |  |  |  |
|  | 120 to 180 minutes | 15.16% | 93.80% | 6.20% | 91.30% | 8.70% |  |  |  |  |
|  | Greater than 180 minutes | 38.84% | 91.10% | 8.90% | 91.30% | 8.70% |  |  |  |  |
| Child Weekend EMU | 0-120 minutes | 28.80% | 96.20% | 3.80% | 94.70% | 5.30% | 28.93 | <0.001 | 17.98 | <0.001 |
|  | 120 to 240 minutes | 30.75% | 93.20% | 6.80% | 92.30% | 7.70% |  |  |  |  |
|  | 240 to 360 minutes | 17.57% | 92.50% | 7.50% | 92.60% | 7.40% |  |  |  |  |
|  | Greater than 360 minutes | 22.88% | 90.40% | 9.60% | 89.80% | 10.20% |  |  |  |  |

**Table S4: Percentages of Boys in Clinical Range for Depression and Anxiety as a Function of Electronic Media Use**

|  |  |  | **Clinical Depression** | | **Clinical Anxiety** | | **Clinical Depression** | | **Clinical**  **Anxiety** | |
| --- | --- | --- | --- | --- | --- | --- | --- | --- | --- | --- |
|  |  | **% in Screentime Category** | NO | YES | NO | YES | χ2 | χ2 *p* | χ2 | χ2 *p* |
| Parent Weekday EMU | 0-60 minutes | 30.22% | 93.50% | 6.50% | 92.20% | 7.80% | 12.03 | 0.007 | 6.95 | 0.074 |
|  | 60 to 120 minutes | 31.05% | 92.40% | 7.60% | 90.70% | 9.30% |  |  |  |  |
|  | 120 to 180 minutes | 19.32% | 90.00% | 10.00% | 89.00% | 11.00% |  |  |  |  |
|  | Greater than 180 minutes | 19.41% | 87.90% | 12.10% | 87.70% | 12.30% |  |  |  |  |
| Parent Weekend EMU | 0-120 minutes | 23.60% | 93.80% | 6.20% | 92.80% | 7.20% | 17.31 | 0.001 | 16.71 | 0.001 |
|  | 120 to 240 minutes | 40.43% | 92.50% | 7.50% | 90.70% | 9.30% |  |  |  |  |
|  | 240 to 360 minutes | 23.46% | 90.20% | 9.80% | 90.40% | 9.60% |  |  |  |  |
|  | Greater than 360 minutes | 12.51% | 85.70% | 14.30% | 83.80% | 16.20% |  |  |  |  |
| Child Weekday EMU | 0-60 minutes | 18.22% | 93.70% | 6.30% | 90.40% | 9.60% | 14.64 | 0.002 | 8.61 | 0.035 |
|  | 60 to 120 minutes | 22.54% | 93.90% | 6.10% | 93.50% | 6.50% |  |  |  |  |
|  | 120 to 180 minutes | 15.50% | 92.30% | 7.70% | 89.90% | 10.10% |  |  |  |  |
|  | Greater than 180 minutes | 43.74% | 88.90% | 11.10% | 88.60% | 11.40% |  |  |  |  |
| Child Weekend EMU | 0-120 minutes | 23.18% | 95.60% | 4.40% | 93.80% | 6.20% | 20.73 | <0.001 | 18.86 | <0.001 |
|  | 120 to 240 minutes | 29.53% | 91.60% | 8.40% | 90.30% | 9.70% |  |  |  |  |
|  | 240 to 360 minutes | 19.83% | 91.00% | 9.00% | 91.40% | 8.60% |  |  |  |  |
|  | Greater than 360 minutes | 27.46% | 87.90% | 12.10% | 86.30% | 13.70% |  |  |  |  |

**Table S5: Percentages of Girls in Clinical Range for Depression and Anxiety as a Function of Electronic Media Use**

|  |  |  | **Clinical Depression** | | **Clinical Anxiety** | | **Clinical Depression** | | **Clinical**  **Anxiety** | |
| --- | --- | --- | --- | --- | --- | --- | --- | --- | --- | --- |
|  |  | **% in Screentime Category** | NO | YES | NO | YES | χ2 | χ2 *p* | χ2 | χ2 *p* |
| Parent Weekday EMU | 0-60 minutes | 34.11% | 96.90% | 3.10% | 96.40% | 3.60% | 10.29 | 0.016 | 11.37 | 0.010 |
|  | 60 to 120 minutes | 32.80% | 95.80% | 4.20% | 94.50% | 5.50% |  |  |  |  |
|  | 120 to 180 minutes | 17.05% | 93.20% | 6.80% | 91.70% | 8.30% |  |  |  |  |
|  | Greater than 180 minutes | 16.04% | 93.40% | 6.60% | 95.90% | 4.10% |  |  |  |  |
| Parent Weekend EMU | 0-120 minutes | 29.36% | 96.90% | 3.10% | 95.70% | 4.30% | 12.76 | 0.005 | 3.35 | 0.340 |
|  | 120 to 240 minutes | 43.34% | 95.10% | 4.90% | 95.20% | 4.80% |  |  |  |  |
|  | 240 to 360 minutes | 18.92% | 95.70% | 4.30% | 93.90% | 6.10% |  |  |  |  |
|  | Greater than 360 minutes | 8.38% | 90.40% | 9.60% | 92.80% | 7.20% |  |  |  |  |
| Child Weekday EMU | 0-60 minutes | 25.98% | 96.50% | 3.50% | 95.50% | 4.50% | 3.36 | 0.339 | 3.21 | 0.360 |
|  | 60 to 120 minutes | 25.78% | 95.50% | 4.50% | 95.30% | 4.70% |  |  |  |  |
|  | 120 to 180 minutes | 14.78% | 95.60% | 4.40% | 92.80% | 7.20% |  |  |  |  |
|  | Greater than 180 minutes | 33.45% | 94.30% | 5.70% | 95.00% | 5.00% |  |  |  |  |
| Child Weekend EMU | 0-120 minutes | 34.96% | 96.50% | 3.50% | 95.20% | 4.80% | 3.37 | 0.339 | 1.33 | 0.721 |
|  | 120 to 240 minutes | 32.09% | 94.80% | 5.20% | 94.30% | 5.70% |  |  |  |  |
|  | 240 to 360 minutes | 15.09% | 94.60% | 5.40% | 94.30% | 5.70% |  |  |  |  |
|  | Greater than 360 minutes | 17.86% | 94.60% | 5.40% | 95.80% | 4.20% |  |  |  |  |
